# Timing of spermarche and menarche among urban students in Guangzhou, China: trends from 2005 to 2012 and association with Obesity

**DOI:** 10.1038/s41598-017-18423-6

**Published:** 2018-01-10

**Authors:** Yusheng Deng, Jianping Liang, Yinan Zong, Ping Yu, Runsheng Xie, Yangfeng Guo, Zhen Wang, Nali Deng, Yanhui Gao, Yi Jiang, Yi Yang, Jiewen Yang, Li Liu

**Affiliations:** 1 Department of Epidemiology and Biostatistics, School of Public Health, Guangdong Pharmaceutical University, Guangzhou, China; 2 Guangzhou Health Care Promotion Center for Primary and Middle Schools, Guangzhou, China

## Abstract

In Guangzhou, China, whether the trend of a decreasing pubertal age has continued in recent years remained unknown, and the association between obesity and early puberty was still controversial. Herein, we conducted a serial cross-sectional study using data from physical fitness surveillance (2005–2012), to determine the recent trends in age at spermarche and menarche among students in Guangzhou, and to investigate whether elevated BMI modified timing of spermarche and menarche. This study included 1,278,258 urban students. In boys, no significant differences were observed in median ages of spermarche (MAS) from 2005 to 2012, with overlapping 95% CIs. Similar results were observed for median ages of menarche (MAM) in girls. The Cox-Stuart trend test showed neither upward nor downward shift in MAS and MAM over time (*P* = 0.625; 1.000). Each year, both MAS and MAM decreased with increasing BMI. Furthermore, a higher BMI was associated with early age at spermarche and menarche, with ORs of 1.052 (95% CI = 1.045–1.059) and 1.233 (95% CI = 1.220–1.247) in 2012 for boys and girls, respectively. In conclusion, the pubertal timing has been stable in urban students from 2005 to 2012. Furthermore, obesity was associated with early timing of spermarche and menarche.

## Introduction

Puberty is a dynamic biological process that involves the exertion of positive feedback on the hypothalamic-pituitary-gonadal (HPG) axis^1^. The physical changes that occur during puberty affect body size, shape and composition and lead to an acquisition of secondary sex characteristics and maturation of reproductive organs. Sex hormones, namely, testosterone in males and estrogen in females, are responsible for these physical changes, which include breast development, the appearance of pubic hair, gonadarche, menarche, and spermarche^2^.

Although puberty is a typical phase of development in adolescents, the timing of puberty varies substantially among individuals^3^. Notably, pubertal timing has been associated with a number of health outcomes. Adolescents with early puberty are more susceptibility to several adverse behaviors, including alcohol abuse, smoking, drug use, early sexual debut^4^, and eating disorders^5^. Additionally, adolescents who mature early are at high risk of developing metabolic syndrome, cardiovascular disease, and reproductive cancers later in adulthood^6–9^. Therefore, a better understanding of the modifiable factors influencing pubertal timing could have important public health implications.

Puberty begins in girls at an average age of 10–11 years and in boys around ages 11–12 years^10^. More importantly, epidemiological evidence has linked variability in pubertal timing to genetics, psychological factors, some environmental chemicals, physical activity, and nutritional status. Of these factors, nutritional influences, as reflected in body size during childhood, are the most relevant^3^. The age of puberty has declined continuously in various populations since the 20^th^ century, while childhood obesity has increased year by year^11,12^. These trends support the potential link between childhood obesity and timing ofpuberty. In girls, the association of higher adiposity with early menarche has been consistently shown. For example, Lee *et al*. suggested that higher body mass index (BMI) z-scores at 3 years of age and rapid weight gain during childhood were positively associated with early menarcheal age^13^. However, studies on the nutritional determinants of pubertal timing in boys remain scarce, and even contradictory results have been reported. Some studies found that obesity was associated with delayed spermarche^14,15^, whereas others found advanced puberty in boys with higher BMI^16–18^. Additional investigations in boys are thus needed to clarify the relationship between nutritional influence and timing of puberty.

Guangzhou is the capital of Guangdong Province and is the leading commercial and largest city in Southern China. According to the 2010 Census estimates, there were 1,919,981 school-aged students, and 82.34% of students were urban residents. Previous publications have reported the possible trends in ages at spermarche and menarche among Guangzhou students, but with inconsistent results. For instance, Mai suggested that the changes in pubertal timing was not obvious between 1985 and 2000^19^, whereas Liang *et al*. showed a decreasing trend in ages at spermarche and menarche in four- or five-year intervals during 1991–2014 based on small sample size^20^. In the report by Liang *et al*., difference in pubertal timing was suggested between urban and rural students, but the difference seems to be gradually narrowing. Additionally, the relationship between obesity and ages at spermarche and menarche in Guangzhou students is still unknown. Accordingly, the current study analyzed data from annual physical fitness surveillance of a large sample of students from 2005 to 2012, to depict the trends in timing of spermarche and menarche in Guangzhou and, more importantly, to explore the possible associations between BMI and ages at spermarche/menarche.

## Methods

### Data/Subjects

Data from the 2005–2012 annual physical fitness surveillance for school-aged students in Guangzhou were collected. The Guangzhou Health Care Promotion Center for Primary and Middle Schools conducted the physical fitness surveillance system, according to the “Implementation Plan of Chinese Students’ Constitution and Health”. We collected eight waves of cross-sectional data on height, weight, and spermarcheal or menarcheal status for all students aged 9–15 years (2005 to 2012). The sample sizes of each age-specific subgroup for the different years ranged from 9,572 to 44,110, with a total of 1,278,258 students included. The age and sex distributions of the subjects are shown in Supplementary Table S1. This study was approved by the institutional review board of Guangdong Pharmaceutical University, and all subjects provided verbal informed consent before the physical examination and interview. All methods in this study was performed in line with the principles of the Declaration of Helsinki, and in accordance with all relevant guidelines and regulations.

### Measures

Well-trained physicians collected information on subjects’ spermarcheal/menarcheal status using the status quo method. All subjects were asked whether they had experienced their first ejaculation or menstruation, and a dichotomous response (yes/no) was obtained for spermarcheal/menarcheal status. The physicians provided sufficient explanations to subjects who did not understand spermarche or menarche.

A portable stadiometer was used to measure height to the nearest 0.1 cm, with subjects in a standing position and without shoes. Weight was measured to the nearest 0.1 kg using a standardized scale with subjects barefoot and in light clothing only. All survey sites applied similar procedures to measure students’ height and weight. Both the scales and stadiometers were calibrated before use. In the physical fitness surveillance, 3% of students were randomly selected to re-examine and re-interview, according to the “Implementation Plan of Chinese Students’ Constitution and Health”. BMI was calculated as weight in kilograms divided by height in meters squared. BMI values were categorized into underweight, normal-weight, overweight, and obesity, according to the sex-age-specific BMI references recommended by the Chinese Working Group on Obesity (WGOC)^21^ and the screening standards for malnutrition of school-age children and adolescents outlined by the National Health and Family Planning Commission of China (NHFPC)^22^. In the WGOC-BMI criteria, children and adolescents with BMI values greater than or equal to the 85th and 95th percentile of the age-sex-specific BMI are defined as overweight and obesity, respectively. For instance, among boys aged 9 years, BMI of 14.1, 18.9 and 21.4 kg/m^2^ are the cutoffs for underweight, overweight and obesity, respectively, while for girls aged 9 years, BMI of 13.8, 19.0 and 21.0 kg/m^2^ are the cutoffs for underweight, overweight and obesity, respectively.

### Statistical analyses

All analyses were separately conducted for boys and girls. The rates of spermarche/menarche by age were calculated for each year. The median ages of spermarche (MAS) and menarche (MAM) and their 95% confidence intervals (CIs) were then calculated according to BMI levels using probit regression analysis^17,23^. The MAS and MAM were defined as the age at which 50% of students were predicted to have reached spermarche or menarche, respectively. Potential trends in MAS and MAM from 2005 to 2012 were explored using the Cox-Stuart test, which tests the null hypothesis for the absence of a trend against the alternative hypothesis of an existing trend independent of the trend distribution^24^. To further investigate the association between BMI and the log odds of reaching spermarche/menarche, logistic regression models were applied after adjusting for students’ age and districts of schools. All statistical analyses were performed using SAS version 9.4 (SAS Institute Inc., Cary, NC., USA) and R V3.1.2 (The R Foundation for Statistical Computing) software. The level of statistical significance was set to alpha < 0.05.

## Results

### Reported rates of spermarche in boys and menarche in girls from 2005 to 2012

Supplementary Table S1 showed the demographic characteristics of the study population from 2005 to 2012. The age and district distributions of the samples among boys and girls were similar. In 2012, the reported rates of spermarche among boys aged 9–11 years were less than or equal to 1.02%. The peak period of spermarche occurred from 12 to 14 years of age, during which the reported rate of spermarche rose from 16.38% to 86.31%. For girls, the peak period of menarche in 2012 ranged from 11 to 13 years of age, with the menarcheal reported rate increasing from 25.27% to 91.58% (Supplementary Table S2). Similar peak ages of spermarche and menarche were observed in other years.

**Figure 1:**
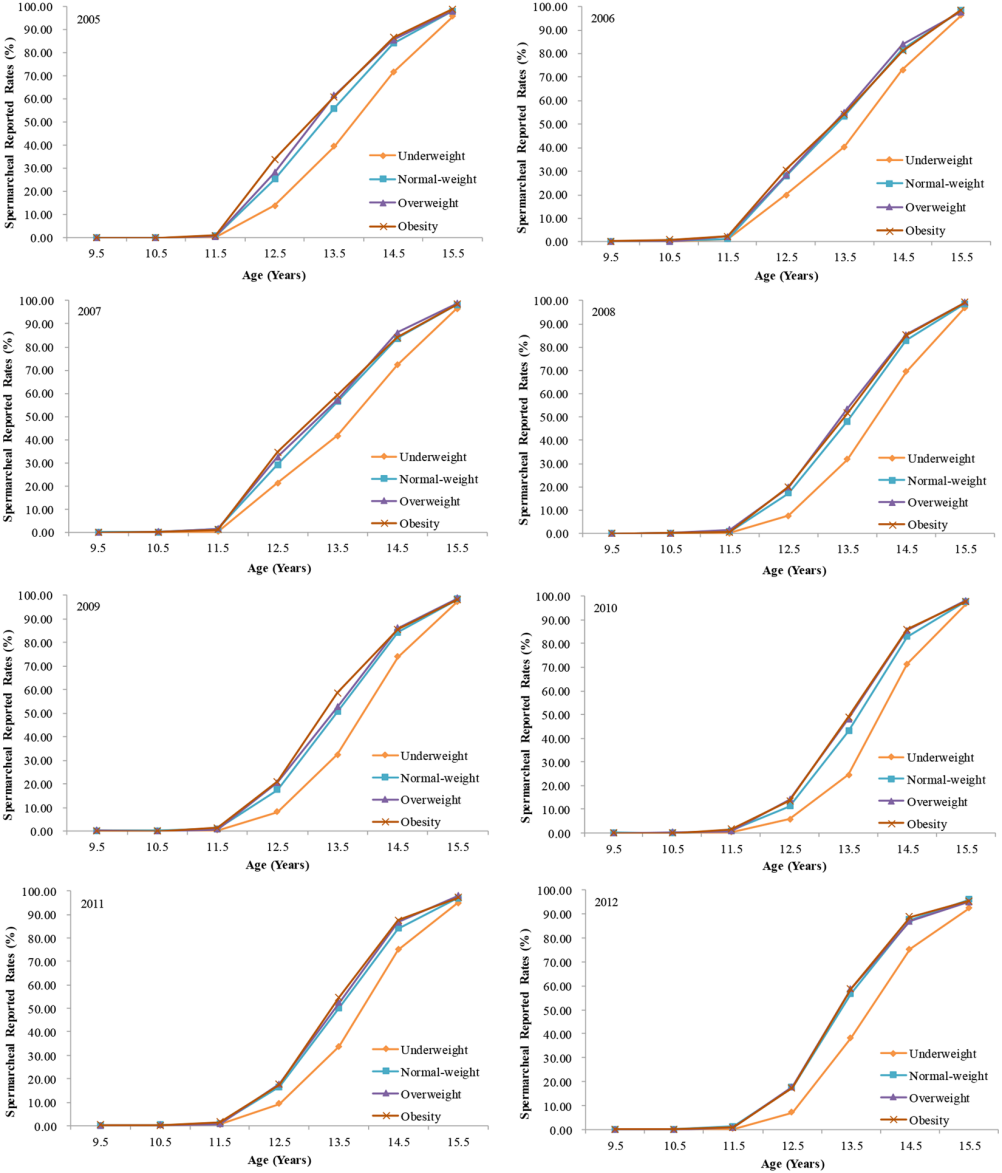
The reported rates of spermarche among boys aged 9–15 years by different BMI levels from 2005 to 2012, Guangzhou, China.

**Figure 2:**
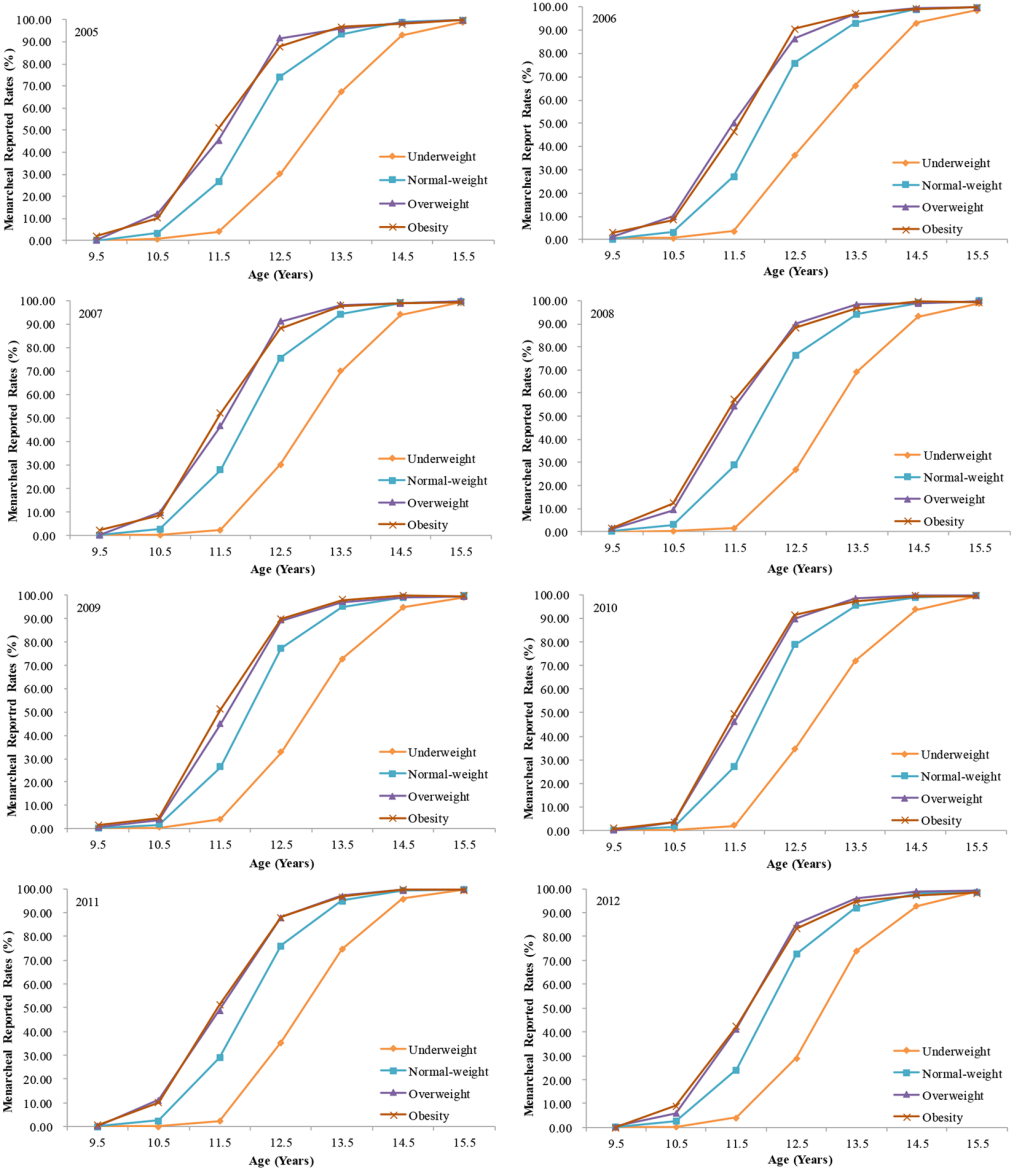
The reported rates of menarche among girls aged 9–15 years by different BMI levels from 2005 to 2012, Guangzhou, China.

More importantly, in the same age group, differences in the reported rates of spermarche and menarche were observed among students with different BMI levels. From 2005 to 2012, the general trends in spermarcheal report rates each year by BMI were as follows: underweight < normal-weight < overweight and obesity (Fig. 1, Supplementary Table S3). In girls, similar trends with changes in BMI were also observed in menarcheal report rates (Fig. 2, Supplementary Table S3).

### Trends in MAS for boys and MAM for girls from 2005 to 2012

**Figure 3:**
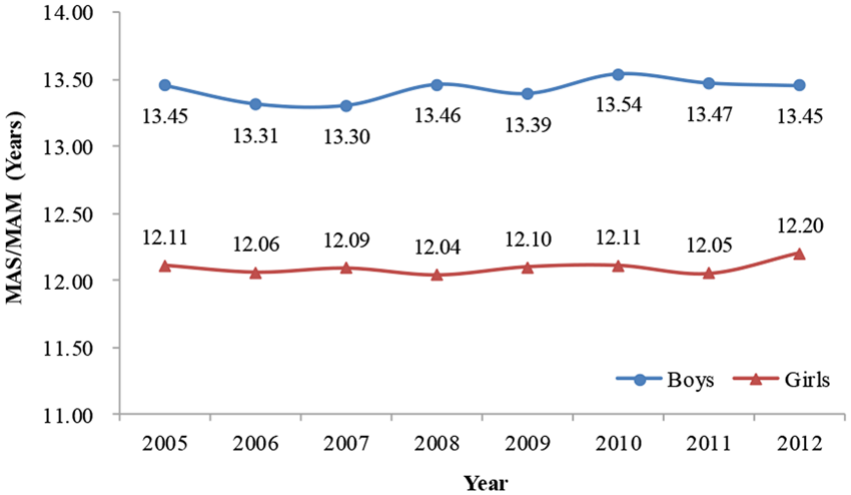
The trends in median ages of spermarche (MAS) for boys and median ages of menarche (MAM) for girls aged 9–15 years from 2005 to 2012, Guangzhou, China.

The MAS in boys was 13.45 years in both 2005 and 2012 (95% CIs = 13.43–13.47 and 13.42–13.48, respectively), while the MAM for girls was 12.11 (95% CI = 12.08–12.14) and 12.20 years (95% CI = 12.17–12.24), respectively. The 95% CIs of MAS in boys and MAM in girls overlapped, suggesting no significant differences in ages at spermarche and menarche between 2005 and 2012 (Supplementary Table S2). As shown in Fig. 3, the MAS in boys and MAM in girls exhibited a stable pattern from 2005–2012. The Cox-Stuart test also showed neither an upward nor downward trend in MAS and MAM over time (MAS: *K* = 3, *P* = 0.625; MAM: *K* = 2, *P* = 1.000). Additionally, the MAS in boys was later than the MAM in girls, consistent with the principles of pubertal development.

**Figure 4:**
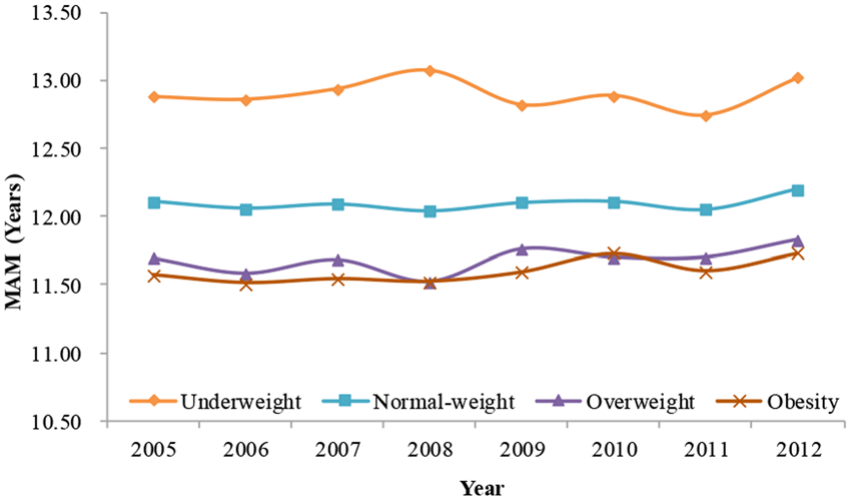
The trends in median ages of menarche (MAM) in girls by different BMI levels from 2005 to 2012, Guangzhou, China.

**Figure 5:**
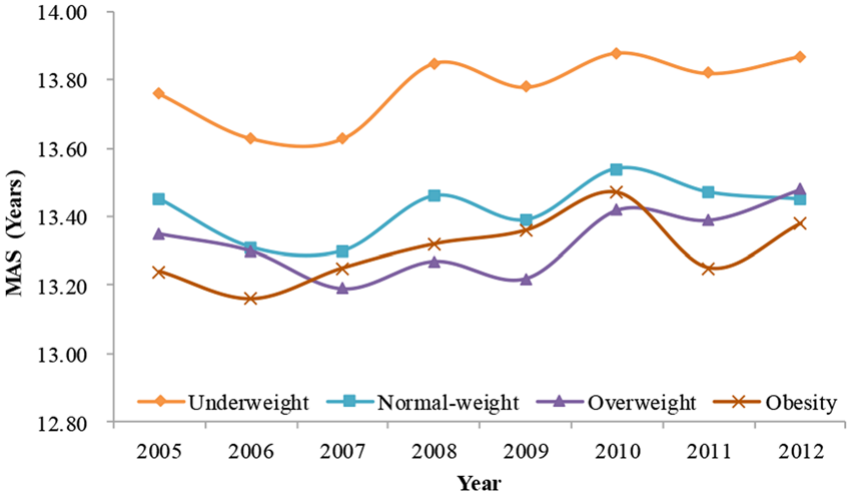
The trends in median ages of spermarche (MAS) in boys by different BMI levels from 2005 to 2012, Guangzhou, China.

Supplementary Table S4 showed the MAS and MAM and their 95% CIs in different BMI groups. The MAM decreased as BMI increased each year (Fig. 4, Supplementary Table S4). The MAM among girls in the underweight, normal-weight, overweight and obesity groups in 2005 were 12.88 (95% CI = 12.79–12.97), 12.11 (95% CI = 12.02–12.09), 11.69 (95% CI = 11.52–11.85) and 11.57 (95% CI = 11.35–11.79) years, respectively, while in 2012, these ages were 13.02 (95% CI = 12.90–13.13), 12.20 (95% CI = 12.16–12.23), 11.83 (95% CI = 11.71–11.97) and 11.73 (95% CI = 11.54–11.93) years, respectively (Fig. 4, Supplementary Table S4). Generally, the MAS of boys also decreased as BMI increased each year (Fig. 5, Supplementary Table S4), but there were overlaps for the 95% CIs of MAS among different BMI groups in several different times.

### Association between BMI and spermarcheal/menarcheal status

A higher BMI conferred a significantly increased likelihood of reaching spermarche for boys and menarche for girls (Table 1). After adjusting for students’ age and districts of schools, the ORs for the association of BMI with spermarche and menarche in 2005 were 1.083 (95% CI = 1.075–1.090) and 1.384 (95% CI = 1.365–1.403), respectively. Similar associations were observed in other years, for example, in 2012, with ORs of 1.052 (95% CI = 1.045–1.059) and 1.233 (95% CI = 1.220–1.247) for boys and girls, respectively. Additionally, when BMI was categorized, after adjusting for students’ age and districts of schools, overweight and obesity both increased the likelihood of reaching menarche for girls in each year (Supplementary Table S5), as compared to the normal-weight. In boys, similar results were observed, except in 2006 and 2012. Of note, BMI presented a stronger association with menarche in girls than with spermarche in boys.

**Table 1:** Logistic regression analysis of BMI and spermarcheal/menarcheal status from 2005 to 2012.

Year	Spermarche		Menarche	
	OR (95% CI)	OR (95% CI)*	OR (95% CI)	OR (95% CI)*
2005	1.175 (1.169–1.180)	1.083 (1.075–1.090)	1.630 (1.614–1.645)	1.384 (1.365–1.403)
2006	1.140 (1.135–1.145)	1.047 (1.041–1.053)	1.618 (1.603–1.633)	1.367 (1.350–1.384)
2007	1.143 (1.138–1.148)	1.059 (1.053–1.066)	1.591 (1.576–1.606)	1.385 (1.367–1.404)
2008	1.148 (1.143–1.153)	1.073 (1.066–1.079)	1.604 (1.590–1.619)	1.395 (1.377–1.413)
2009	1.134 (1.129–1.138)	1.066 (1.060–1.072)	1.532 (1.519–1.545)	1.340 (1.322–1.357)
2010	1.131 (1.127–1.136)	1.068 (1.062–1.075)	1.542 (1.529–1.556)	1.330 (1.314–1.347)
2011	1.129 (1.124–1.133)	1.059 (1.053–1.066)	1.550 (1.536–1.563)	1.300 (1.285–1.316)
2012	1.123 (1.119–1.128)	1.052 (1.045–1.059)	1.446 (1.434–1.459)	1.233 (1.220–1.247)

Abbreviations: OR, odds ratio; CI, confidence interval. *Adjusted for students’ age and districts of schools.

## Discussion

This serial cross-sectional study, which analyzed data from eight waves of physical fitness surveillance from 2005 to 2012, suggested that the ages of spermarche and menarche appeared to level off for urban students in Guangzhou, as similar median ages of spermarche (MAS) and menarche (MAS) were shown each year. Furthermore, no significant changes in this 8-year period were indicated by the Cox-Stuart test. The stable trend of MAS and MAM shown in this study is consistent with a previous study by Mai *et al*.^19^ in Guangzhou students. In the other study on pubertal timing of Guangzhou students, as suggested by Liang *et al*.^20^, the decline trends appeared to stop in urban students from 2005 to 2014, in accordance with the stable pattern of pubertal timing found in our study.

In addition to the rural-urban disparity, other factors, such as living conditions, physical activity, and nutrition status, also affect the secular trends in pubertal timing. With the achievement of an optimal level of nutrition and living conditions, pubertal timing has stabilized in some developed regions in the past few decades^25,26^. Genetic effects have also been suggested to account for at least 46% of the variability in pubertal timing^3^. From an evolutionary perspective, the so-called “target-seeking growth model” in physical anthropology indicates that genetic determinants would exert a repressive effect on excessively decreased trends in pubertal timing^27^. Specifically, if the timing of puberty decreased to a certain age, this declining trend would slow or even stop due to genetic factors. Additionally, the MAS and MAM for urban students in Guangzhou were both younger than those of Han students obtained at the national level. For instance, in 2010, the MAM in Guangzhou was 12.11 years, whereas it was 12.47 years in Han girls nationally, suggesting that sex education should start at an earlier age for Guangzhou students.

More importantly, we found that the reported rates of menarche and spermarche increased with increasing BMI levels in the same age group over time, while MAS and MAM both declined as BMI increased. The logistic regression analysis further suggested that higher BMI conferred an increased likelihood of early puberty for both sex, with a greater risk in girls than in boys. Epidemiological evidence has supported the role of body fat on the timing of sexual development. The onset of puberty has been suggested to be triggered by the attainment of a critical amount and distribution of body fat^28^. Rapid weight gain in early life has been related to early puberty in both sex but especially in girls^29^. An association between elevated BMI and earlier puberty has consistently been shown in girls^13,30,31^, whereas in boys, this relationship has been somewhat less consistent^14–18^. However, our results agree with multiple previous studies, which show that the heavier boys are in childhood, the earlier they begin puberty and that boys with underweight thus start puberty later than their peers^32^.

Although the association of BMI with puberty was stronger in girls than in boys, the underlying mechanisms behind the modulation of BMI on pubertal timing in girls and boys may be largely similar. One important factor potentially linking obesity to the timing of puberty is leptin. As an adipokine, leptin signals the state of energy stores to the brain, and elevated serum leptin levels have been reported in children with obesity^33^. Leptin receptors are abundantly expressed in the hypothalamus and anterior pituitary, and leptin has a direct, dose-response effect on the HPG axis by accelerating the secretion of gonadotropin-releasing hormone (GnRH)^34–36^. In normal mice, starvation-induced decreases in leptin have been related to reduced activity of the HPG axis^37^. By contrast, exogenous supplements of leptin have been shown to maintain the activity of the HPG axis and accelerate the onset of puberty in normal mice^38^. Additionally, in leptin-deficient mice, central hypogonadism that can be reversed after leptin treatment has been observed^12,34^. Consistent results regarding the role of leptin in pubertal development have been observed in children^36^. Other hypotheses about the acceleration of puberty by obesity involve insulin-like growth factor, insulin, and adiponectin, which may alter the expression of sex hormone-binding globulin. Nevertheless, the precise mechanisms explaining how excess adiposity promotes the onset of puberty remain unclear, and further research is needed to clearly delineate the effects of elevated BMI on pubertal development.

This study was based on a large sample of data from sequential surveillance of students’ physical fitness; however, some limitations should be mentioned. One limitation is the use of self-reported menarcheal and spermarcheal status. The median ages of menarche and spermarche may not reflect the exact timing of puberty. However, in population-based studies, the status quo method has been considered to be more reliable than recall for dates of menarche/spermarche. Second, the rural-urban differences in the trends in pubertal timing were not investigated in this study, because of the lack of available data for rural students. Third, although the development of puberty is influenced by multiple factors, we only investigate the association between adiposity and timing of puberty after adjusting for students’ age and districts of schools. Other potential confounders should be included in the future study with comprehensive design. Finally, this study used a serial cross-sectional design. Although we observed consistent associations between elevated BMI and earlier ages at spermarche and menarche in all data waves, the causal relationship between adiposity and puberty development should be further established by a comprehensive prospective study.

In conclusion, from 2005 to 2012, the downward trend in ages at spermarche and menarche among urban students in Guangzhou stopped and entered a period of stabilization. Nonetheless, whether the downward trend resumed after 2012 in Guangzhou remains uncertain and warrants further longitudinal tracking. Furthermore, this study suggested that elevated BMI was associated with earlier timing of puberty in both girls and boys. Continued research is needed, to thoroughly explore nutritional determinants of pubertal timing and to further develop comprehensive nutritional interventions in management of early puberty.

## Electronic supplementary material

Supplementary Information
