# Supplementary material for: Timing of spermarche and menarche among urban students in Guangzhou, China: trends from 2005 to 2012 and association with Obesity
Source: Sci Rep. 2018 Jan 10;8:263. doi: 10.1038/s41598-017-18423-6 (PMC5762656; doi:10.1038/s41598-017-18423-6)
Supplement: Supplementary file 1 — Supplementary Information [file 41598_2017_18423_MOESM1_ESM.pdf]

Timing of spermarche and menarche among urban students in Guangzhou, China: trends from 2005 to 2012 and association with Obesity

Yusheng Deng[1], Jianping Liang[2], Yinan Zong[1], Ping Yu[2], Runsheng Xie[1], Yangfeng Guo[2], Zhen Wang[1], Nali Deng[2], Yanhui Gao[1], Yi Jiang[2], Yi Yang[1], Jiewen Yang[2*], & Li Liu[1*]

Y. Deng and J. Liang contributed equally to this work.

[1] Department of Epidemiology and Biostatistics, School of Public Health, Guangdong Pharmaceutical University, Guangzhou, China

[2] Guangzhou Health Care Promotion Center for Primary and Middle Schools, Guangzhou, China.

[*]Corresponding author:

L. Liu, Department of Epidemiology and Biostatistics, School of Public Health, Guangdong Pharmaceutical University, Guangzhou 510310, China; Phone: 86-20-34055123; FAX: 86-20-34055355; E-mail: pupuliu919@163.com.

J. Yang, Guangzhou Health Care Promotion Center for Primary and Middle Schools, Guangzhou 510180, China; Phone: 86-020-81079384; E-mail: 360449586@qq.com.

Table S1 The demographic characteristics of students from 2005 to 2012 in Guangzhou, China.

	2005 N=152,764	2006 N=168,422	2007 N=159,309	2008 N=164,121	2009 N=161,755	2010 N=167,691	2011 N=166,261	2012 N=137,935
**Boys**								
**Age (years)**								
**9~**	5,719 (7.22)	5,833 (6.66)	5,862 (7.03)	6,232 (7.25)	6,635 (7.85)	6,007 (6.84)	6,059 (6.94)	5,197 (7.23)
**10~**	5,614 (7.09)	5,931 (6.77)	6,150 (7.38)	6,228 (7.25)	6,057 (7.17)	6,871 (7.83)	6,078 (6.96)	5,186 (7.21)
**11~**	5,550 (7.01)	5,819 (6.64)	6,174 (7.41)	6,589 (7.67)	6,326 (7.49)	6,509 (7.42)	7,056 (8.08)	5,482 (7.62)
**12~**	9,472 (11.96)	10,937 (12.48)	11,688 (14.02)	12,670 (14.75)	12,218 (14.46)	13,059 (14.88)	12,905 (14.78)	13,087 (18.20)
**13~**	16,372 (20.68)	18,787 (21.44)	17,357 (20.82)	17,824 (20.75)	18,263 (21.62)	18,174 (20.71)	18,566 (21.26)	14,119 (19.63)
**14~**	16,326 (20.62)	17,862 (20.39)	17,335 (20.80)	17,406 (20.26)	17,002 (20.13)	18,779 (21.39)	18,273 (20.92)	15,006 (20.87)
**15~16**	20,124 (25.42)	22,451 (25.62)	18,786 (22.54)	18,967 (22.08)	17,974 (21.28)	18,374 (20.93)	18,396 (21.06)	13,842 (19.25)
**District**								
**Baiyun**	12,486 (15.77)	14,445 (16.49)	14,145 (16.97)	15,041 (17.51)	14,511 (17.18)	15,697 (17.88)	14,572 (16.69)	11,073 (15.4)
**Haizhu**	20,873 (26.36)	21,613 (24.67)	20,579 (24.69)	20,761 (24.16)	20,711 (24.52)	20,933 (23.85)	21,349 (24.45)	13,971 (19.43)
**Huangpu**	2,974 (3.76)	2,971 (3.39)	2,475 (2.97)	2,737 (3.19)	2,684 (3.18)	3,168 (3.61)	3,251 (3.72)	3,477 (4.83)
**Liwan**	13,332 (16.84)	14,864 (16.96)	13,369 (16.04)	12,870 (14.98)	13,183 (15.61)	13,288 (15.14)	13,112 (15.01)	9,450 (13.14)
**Luogang**	616 (0.78)	1,218 (1.39)	1,511 (1.81)	2,392 (2.78)	2,507 (2.97)	2,676 (3.05)	2,778 (3.18)	2,772 (3.85)
**Panyu**	1,614 (2.04)	1,340 (1.53)	1,627 (1.95)	1,870 (2.18)	1,632 (1.93)	1,896 (2.16)	2,071 (2.37)	2,384 (3.31)
**Tianhe**	4,278 (5.40)	4,525 (5.16)	4,426 (5.31)	4,949 (5.76)	4,786 (5.67)	4,972 (5.66)	4,893 (5.60)	5,322 (7.40)
**Yuexiu**	21,401 (27.03)	24,253 (27.68)	22,755 (27.30)	22,706 (26.43)	21,974 (26.01)	21,974 (25.04)	21,771 (24.93)	19,687 (27.37)
**Others**	1,603 (2.02)	2,391 (2.73)	2,465 (2.96)	2,590 (3.01)	2,487 (2.95)	3,169 (3.61)	3,536 (4.04)	3,783 (5.26)
**BMI levels**								
**Normal-weight**	52,748 (66.62)	58,351 (66.60)	55,719 (66.85)	57,959 (67.46)	55,852 (66.12)	58,487 (66.63)	57,919 (66.32)	47,356 (65.85)
**Underweight**	13,385 (16.91)	14,480 (16.53)	13,359 (16.03)	11,934 (13.89)	11,291 (13.37)	11,207 (12.77)	10,819 (12.39)	7,796 (10.84)
**Overweight**	8,663 (10.94)	9,789 (11.17)	9,430 (11.31)	10,420 (12.13)	11,045 (13.07)	11,451 (13.05)	11,873 (13.6)	10,713 (14.90)
**Obesity**	4,381 (5.53)	5,000 (5.71)	4,844 (5.81)	5,603 (6.52)	6,287 (7.44)	6,628 (7.55)	6,722 (7.70)	6,054 (8.42)
**Girls**								
**Age (years)**								
**9~**	5,012 (6.81)	4,834 (5.98)	4,847 (6.38)	5,359 (6.85)	5,501 (7.12)	4,973 (6.22)	5,085 (6.44)	4,375 (6.63)
**10~**	4,755 (6.46)	5,075 (6.28)	4,968 (6.54)	5,197 (6.65)	5,285 (6.84)	5,762 (7.21)	5,067 (6.42)	4,416 (6.69)
**11~**	4,898 (6.66)	4,936 (6.11)	5,498 (7.24)	5,513 (7.05)	5,309 (6.87)	5,850 (7.32)	5,979 (7.58)	4,836 (7.33)

**12~**	9,123 (12.40)	10,421 (12.90)	10,802 (14.22)	11,842 (15.14)	11,300 (14.62)	11,480 (14.36)	11,986 (15.19)	11,984 (18.15)
**13~**	15,196 (20.65)	17,484 (21.64)	16,143 (21.25)	16,173 (20.68)	16,470 (21.31)	16,220 (20.30)	16,014 (20.29)	13,011 (19.71)
**14~**	15,116 (20.54)	16,393 (20.29)	15,822 (20.83)	15,835 (20.25)	15,393 (19.92)	17,122 (21.42)	16,574 (21.00)	13,125 (19.88)
**15~16**	19,487 (26.48)	21,659 (26.81)	17,877 (23.54)	18,286 (23.38)	18,022 (23.32)	18,511 (23.16)	18,223 (23.09)	14,269 (21.61)
**District**								
**Baiyun**	11,175 (15.19)	12,973 (16.06)	12,120 (15.96)	12,747 (16.30)	12,313 (15.93)	13,332 (16.68)	12,590 (15.95)	9,507 (14.40)
**Haizhu**	19,298 (26.22)	19,601 (24.26)	18,420 (24.25)	18,542 (23.71)	18,471 (23.90)	18,314 (22.92)	18,591 (23.55)	12,180 (18.45)
**Huangpu**	2,818 (3.83)	2,675 (3.31)	2,253 (2.97)	2,455 (3.14)	2,481 (3.21)	2,960 (3.70)	2,953 (3.74)	3,106 (4.70)
**Liwan**	12,938 (17.58)	14,211 (17.59)	12,664 (16.67)	12,218 (15.62)	12,671 (16.40)	12,835 (16.06)	12,540 (15.89)	9,498 (14.39)
**Luogang**	527 (0.72)	980 (1.21)	1,465 (1.93)	2,217 (2.83)	2,398 (3.10)	2,435 (3.05)	2,369 (3.00)	2,420 (3.67)
**Panyu**	1,458 (1.98)	1,168 (1.45)	1,434 (1.89)	1,664 (2.13)	1,373 (1.78)	1,419 (1.78)	1,522 (1.93)	1,842 (2.79)
**Tianhe**	3,767 (5.12)	4,012 (4.97)	3,958 (5.21)	4,391 (5.61)	4,247 (5.50)	4,401 (5.51)	4,267 (5.41)	4,602 (6.97)
**Yuexiu**	19,910 (27.06)	22,559 (27.92)	20,933 (27.56)	21,152 (27.05)	20,611 (26.67)	20,863 (26.11)	20,276 (25.69)	18,690 (28.31)
**Others**	1,696 (2.30)	2,623 (3.25)	2,710 (3.57)	2,819 (3.60)	2,715 (3.51)	3,359 (4.20)	3,820 (4.84)	4,171 (6.32)
**BMI levels**								
**Normal-weight**	58,009 (78.83)	64,549 (79.89)	61,107 (80.45)	63,124 (80.72)	61,940 (80.15)	64,349 (80.52)	63,433 (80.37)	53,214 (80.61)
**Underweight**	9,043 (12.29)	8,454 (10.46)	7,586 (9.99)	6,799 (8.69)	7,018 (9.08)	6,624 (8.29)	6,532 (8.28)	4,661 (7.06)
**Overweight**	4,400 (5.98)	5,167 (6.39)	4,742 (6.24)	5,485 (7.01)	5,398 (6.98)	5,898 (7.38)	5,919 (7.50)	5,427 (8.22)
**Obesity**	2,135 (2.90)	2,632 (3.26)	2,522 (3.32)	2,797 (3.58)	2,924 (3.78)	3,047 (3.81)	3,044 (3.86)	2,714 (4.11)
**All**								
**Age (years)**								
**9~**	10,731 (7.02)	10,667 (6.33)	10,709 (6.72)	11,591 (7.06)	12,136 (7.50)	10,980 (6.55)	11,144 (6.70)	9,572 (6.94)
**10~**	10,369 (6.79)	11,006 (6.53)	11,118 (6.98)	11,425 (6.96)	11,342 (7.01)	12,633 (7.53)	11,145 (6.70)	9,602 (6.96)
**11~**	10,448 (6.84)	10,755 (6.39)	11,672 (7.33)	12,102 (7.37)	11,635 (7.19)	12,359 (7.37)	13,035 (7.84)	10,318 (7.48)
**12~**	18,595 (12.17)	21,358 (12.68)	22,490 (14.12)	24,512 (14.94)	23,518 (14.54)	24,539 (14.63)	24,891 (14.97)	25,071 (18.18)
**13~**	31,568 (20.66)	36,271 (21.54)	33,500 (21.03)	33,997 (20.71)	34,733 (21.47)	34,394 (20.51)	34,580 (20.80)	27,130 (19.67)
**14~**	31,442 (20.58)	34,255 (20.34)	33,157 (20.81)	33,241 (20.25)	32,395 (20.03)	35,901 (21.41)	34,847 (20.96)	28,131 (20.39)
**15~16**	39,611 (25.93)	44,110 (26.19)	36,663 (23.01)	37,253 (22.70)	35,996 (22.25)	36,885 (22.00)	36,619 (22.03)	28,111 (20.38)
**District**								
**Baiyun**	23,661 (15.49)	27,418 (16.28)	26,265 (16.49)	27,788 (16.93)	26,824 (16.58)	29,029 (17.31)	27,162 (16.34)	20,580 (14.92)
**Haizhu**	40,171 (26.30)	41,214 (24.47)	38,999 (24.48)	39,303 (23.95)	39,182 (24.22)	39,247 (23.40)	39,940 (24.02)	26,151 (18.96)
**Huangpu**	5,792 (3.79)	5,646 (3.35)	4,728 (2.97)	5,192 (3.16)	5,165 (3.19)	6,128 (3.65)	6,204 (3.73)	6,583 (4.77)

**Liwan**	26,270 (17.20)	29,075 (17.26)	26,033 (16.34)	25,088 (15.29)	25,854 (15.98)	26,123 (15.58)	25,652 (15.43)	18,948 (13.74)
**Luogang**	1,143 (0.75)	2,198 (1.31)	2,976 (1.87)	4,609 (2.81)	4,905 (3.03)	5,111 (3.05)	5,147 (3.10)	5,192 (3.76)
**Panyu**	3,072 (2.01)	2,508 (1.49)	3,061 (1.92)	3,534 (2.15)	3,005 (1.86)	3,315 (1.98)	3,593 (2.16)	4,226 (3.06)
**Tianhe**	8,045 (5.27)	8,537 (5.07)	8,384 (5.26)	9,340 (5.69)	9,033 (5.58)	9,373 (5.59)	9,160 (5.51)	9,924 (7.19)
**Yuexiu**	41,311 (27.04)	46,812 (27.79)	43,688 (27.42)	43,858 (26.72)	42,585 (26.33)	42,837 (25.55)	42,047 (25.29)	38,377 (27.82)
**Others**	3,299 (2.16)	5,014 (2.98)	5,175 (3.25)	5,409 (3.30)	5,202 (3.22)	6,528 (3.89)	7,356 (4.42)	7,954 (5.77)
**BMI levels**								
**Normal-weight**	110,757 (72.5)	122,900 (72.97)	116,826 (73.33)	121,083 (73.78)	117,792 (72.82)	122,836 (73.25)	12,1352 (72.99)	100,570 (72.91)
**Underweight**	22,428 (14.68)	22,934 (13.62)	20,945 (13.15)	18,733 (11.41)	18,309 (11.32)	17,831 (10.63)	17,351 (10.44)	12,457 (9.03)
**Overweight**	13,063 (8.55)	14,956 (8.88)	14,172 (8.90)	15,905 (9.69)	16,443 (10.17)	17,349 (10.35)	17,792 (10.70)	16,140 (11.70)
**Obesity**	6,516 (4.27)	7,632 (4.53)	7,366 (4.62)	8,400 (5.12)	9,211 (5.69)	9,675 (5.77)	9,766 (5.87)	8,768 (6.36)

Table S2 Median Age of Spermarche in boys and Median Age of Menarche in girls aged 9-15 years from 2005 to 2012, Guangzhou, China.

Year	Age (y)	Median Age (y)	Boys			Girls		
			N	Spermarche	%	N	Menarche	%
2005	9~	9.5	5,719	0	0	5,012	14	0.28
	10~	10.5	5,614	1	0.02	4,755	181	3.81
	11~	11.5	5,550	40	0.72	4,898	1,252	25.56
	12~	12.5	9,472	2,306	24.35	9,123	6,530	71.58
	13~	13.5	16,372	8,855	54.09	15,196	13,844	91.10
	14~	14.5	16,326	13,417	82.18	15,116	14,839	98.17
	15~16	15.5	20,124	19,669	97.74	19,487	19,423	99.67
	Median Age			13.45			12.11	
	95% PI			11.72-15.44			10.22-14.35	
	95% CI			13.43-13.47			12.08-12.14	
2006	9~	9.5	5,833	3	0.05	4,834	27	0.56
	10~	10.5	5,931	16	0.27	5,075	173	3.41
	11~	11.5	5,819	90	1.55	4,936	1,312	26.58
	12~	12.5	10,937	2,935	26.84	10,421	7,672	73.62
	13~	13.5	18,787	9,650	51.37	17,484	15,924	91.08
	14~	14.5	17,862	14,380	80.51	16,393	16,134	98.42
	15~16	15.5	22,451	21,991	97.95	21,659	21,577	99.62
	Median Age			13.31			12.06	
	95% PI			11.16-15.88			10.13-14.35	
	95% CI			13.29-13.34			12.03-12.09	
2007	9~	9.5	5,862	3	0.05	4,847	13	0.27
	10~	10.5	6,150	9	0.15	4,968	151	3.04
	11~	11.5	6,174	75	1.21	5,498	1,505	27.37
	12~	12.5	11,688	3,364	28.78	10,802	7,915	73.27
	13~	13.5	17,357	9,408	54.20	16,143	14,982	92.81
	14~	14.5	17,335	14,203	81.93	15,822	15,598	98.58
	15~16	15.5	18,786	18,412	98.01	17,877	17,794	99.54
	Median Age			13.30			12.09	
	95% PI			11.20-15.80			10.22-14.31	
	95% CI			13.27-13.33			12.06-12.12	
2008	9~	9.5	6,232	0	0	5,359	17	0.32
	10~	10.5	6,228	5	0.08	5,197	187	3.60
	11~	11.5	6,589	54	0.82	5,513	1,611	29.22
	12~	12.5	12,670	2,087	16.47	11,842	8,806	74.36
	13~	13.5	17,824	8,308	46.61	16,173	15,008	92.80
	14~	14.5	17,406	14,155	81.32	15,835	15,599	98.51
	15~16	15.5	18,967	18,677	98.47	18,286	18,235	99.72
	Median Age			13.46			12.04	
	95% PI			11.66-15.53			10.18-14.25	
	95% CI			13.44-13.48			12.01-12.07	
	9~	9.5	6,635	11	0.17	5,501	19	0.35
	10~	10.5	6,057	6	0.10	5,285	90	1.70
	11~	11.5	6,326	49	0.77	5,309	1,418	26.71
	12~	12.5	12,218	2,075	16.98	11,300	8,530	75.49

**2009**	**13~**	**13.5**	18,263	8,930	48.90	16,470	15,449	93.80
	**14~**	**14.5**	17,002	14,148	83.21	15,393	15,199	98.74
	**15~16**	**15.5**	17,974	17,660	98.25	18,022	17,941	99.55
	**Median Age**			13.39			12.10	
	**95% PI**			11.22-15.99			10.25-14.28	
	**95% CI**			13.36-13.42			12.06-12.13	
**2010**	**9~**	**9.5**	6,007	4	0.07	4,973	11	0.22
	**10~**	**10.5**	6,871	3	0.04	5,762	98	1.70
	**11~**	**11.5**	6,509	57	0.88	5,850	1,598	27.32
	**12~**	**12.5**	13,059	1,466	11.23	11,480	8,869	77.26
	**13~**	**13.5**	18,174	7,583	41.72	16,220	15,276	94.18
	**14~**	**14.5**	18,779	15,392	81.96	17,122	16,872	98.54
	**15~16**	**15.5**	18,374	17,957	97.73	18,511	18,428	99.55
	**Median Age**			13.54			12.11	
	**95% PI**			11.40-16.09			10.29-14.26	
	**95% CI**			13.52-13.57			12.08-12.14	
**2011**	**9~**	**9.5**	6,059	5	0.08	5,085	9	0.18
	**10~**	**10.5**	6,078	9	0.15	5,067	164	3.24
	**11~**	**11.5**	7,056	63	0.89	5,979	1,745	29.19
	**12~**	**12.5**	12,905	2,051	15.89	11,986	8,998	75.07
	**13~**	**13.5**	18,566	9,036	48.67	16,014	15,053	94.00
	**14~**	**14.5**	18,273	15,249	83.45	16,574	16,428	99.12
	**15~16**	**15.5**	18,396	17,793	96.72	18,223	18,161	99.66
	**Median Age**			13.47			12.05	
	**95% PI**			11.26-16.11			10.25-14.17	
	**95% CI**			13.44-13.50			12.02-12.07	
**2012**	**9~**	**9.5**	5,197	1	0.02	4,375	12	0.27
	**10~**	**10.5**	5,186	6	0.12	4,416	134	3.03
	**11~**	**11.5**	5,482	56	1.02	4,836	1,222	25.27
	**12~**	**12.5**	13,087	2,144	16.38	11,984	8,611	71.85
	**13~**	**13.5**	14,119	7,788	55.16	13,011	11,916	91.58
	**14~**	**14.5**	15,006	12,951	86.31	13,125	12,813	97.62
	**15~16**	**15.5**	13,842	13,206	95.41	14,269	14,081	98.68
	**Median Age**			13.45			12.20	
	**95% PI**			11.35-15.95			10.21-14.59	
	**95% CI**			13.42-13.48			12.17-12.24	

Abbreviations: CI, confidence interval; MAS, Median Age of Spermarche; MAM, Median Age of Menarche; PI, Prediction Interval

Table S3 The reported rates of spermarche among boys and menarche among girls by different BMI levels from 2005 to 2012, Guangzhou, China.

Year	Gender	Median Age (y)	Underweight		Normal-weight		Overweight		Obesity	
			N	%	N	%	N	%	N	%
2005	Boys	**9.5**	0	0	0	0	0	0	0	0
		**10.5**	0	0	1	0.03	0	0	0	0
		**11.5**	4	0.40	27	0.80	5	0.65	4	1.01
		**12.5**	220	13.82	1,525	25.37	365	28.27	196	33.91
		**13.5**	1,107	39.52	6,105	56.02	1,137	61.66	506	61.04
		**14.5**	2,034	71.72	9,387	84.06	1,372	85.59	624	86.67
		**15.5**	3,221	95.72	13,718	98.08	1,793	98.19	937	98.94
	Girls	**9.5**	2	0.19	7	0.20	1	0.33	4	2.00
		**10.5**	7	0.85	120	3.47	34	12.27	20	10.20
		**11.5**	26	4.08	1,014	26.55	112	45.53	100	51.02
		**12.5**	268	30.15	5,404	74.20	545	91.60	313	87.92
		**13.5**	1,015	67.22	11,567	93.48	868	95.91	394	96.81
		**14.5**	1,584	93.07	12,076	98.85	842	98.71	337	97.97
		**15.5**	2,420	98.86	15,347	99.79	1,221	99.84	435	99.77
2006	Boys	**9.5**	0	0	2	0.05	0	0	1	0.20
		**10.5**	0	0	11	0.30	1	0.13	4	0.78
		**11.5**	12	1.20	48	1.36	20	2.36	10	2.22
		**12.5**	347	19.86	1,931	27.87	428	28.27	229	30.61
		**13.5**	1,337	40.48	6,675	53.40	1,145	55.15	493	54.36
		**14.5**	2,199	73.08	10,023	81.77	1,476	84.05	682	81.19
		**15.5**	3,461	96.27	15,428	98.32	2,087	97.80	1,015	98.45
	Girls	**9.5**	6	0.66	11	0.32	4	1.26	6	2.94
		**10.5**	5	0.61	119	3.18	32	9.94	17	8.50
		**11.5**	21	3.72	1,049	27.09	143	50.18	99	46.26
		**12.5**	326	36.22	6,401	75.79	577	86.25	368	90.64
		**13.5**	1,011	66.30	13,302	93.05	1,056	96.79	555	97.03
		**14.5**	1,447	93.05	13,212	98.95	1,040	99.33	435	99.09
		**15.5**	2,160	98.54	17,388	99.73	1,433	99.86	596	99.83
2007	Boys	**9.5**	1	0.11	2	0.05	0	0	0	0
		**10.5**	1	0.11	4	0.11	3	0.32	1	0.19
		**11.5**	4	0.40	51	1.35	14	1.58	6	1.17
		**12.5**	389	21.26	2,159	29.19	544	32.46	272	34.65
		**13.5**	1,222	41.75	6,537	56.48	1,132	57.09	517	59.15
		**14.5**	2,093	72.17	10,051	83.60	1,373	86.03	686	84.07
		**15.5**	2,841	96.53	13,108	98.21	1,671	98.82	792	98.39
	Girls	**9.5**	1	0.12	6	0.17	1	0.31	5	2.29
		**10.5**	1	0.15	100	2.67	30	9.84	20	8.47
		**11.5**	14	2.27	1,198	27.98	169	46.69	124	52.10
		**12.5**	262	30.15	6,662	75.43	601	91.20	390	88.24
		**13.5**	872	69.93	12,619	94.35	996	98.13	495	97.83
		**14.5**	1,402	94.03	12,767	99.08	978	98.79	451	99.12
		**15.5**	1,860	99.15	14,426	99.57	1,084	99.82	424	99.30
		**9.5**	0	0	0	0	0	0	0	0
		**10.5**	0	0	3	0.08	1	0.11	1	0.17

		**11.5**	2	0.20	33	0.83	16	1.54	3	0.51
	**Boys**	**12.5**	133	7.53	1,390	17.22	379	19.86	185	20.07
		**13.5**	834	31.70	5,806	48.22	1,137	53.51	531	51.70
		**14.5**	1,701	69.37	10,081	82.83	1,574	85.31	799	85.18
		**15.5**	2,444	96.95	13,481	98.59	1,825	99.29	927	99.25
**2008**		**9.5**	0	0	8	0.21	5	1.27	4	1.45
		**10.5**	2	0.32	121	3.06	33	9.27	31	12.25
		**11.5**	8	1.58	1,270	28.82	189	54.15	144	57.14
	**Girls**	**12.5**	219	26.74	7,400	76.33	785	90.02	402	88.16
		**13.5**	804	69.25	12,571	94.21	1,089	98.46	544	96.63
		**14.5**	1,098	93.05	12,902	98.94	1,110	98.75	489	99.59
		**15.5**	1,631	98.85	14,817	99.82	1,284	99.92	503	99.21
		**9.5**	1	0.12	8	0.19	2	0.22	0	0
		**10.5**	1	0.13	5	0.14	0	0	0	0
		**11.5**	2	0.22	32	0.85	7	0.68	8	1.25
	**Boys**	**12.5**	133	8.04	1,336	17.50	393	20.60	213	20.90
		**13.5**	810	32.40	6,180	50.60	1,250	52.70	690	58.62
		**14.5**	1,652	73.88	9,968	84.30	1,621	86.22	907	85.49
		**15.5**	2,340	97.34	12,382	98.35	1,936	98.78	1,002	98.24
**2009**		**9.5**	3	0.33	9	0.23	3	0.67	4	1.30
		**10.5**	2	0.29	60	1.53	15	3.80	13	4.64
		**11.5**	20	3.90	1,097	26.35	162	44.88	139	51.10
	**Girls**	**12.5**	260	32.58	7,087	77.21	728	89.22	455	89.74
		**13.5**	831	72.58	12,970	95.13	1,098	97.17	550	98.04
		**14.5**	1,146	94.95	12,459	99.01	1,089	99.27	505	99.80
		**15.5**	1,756	99.10	14,548	99.60	1,148	99.57	489	99.59
		**9.5**	0	0	4	0.11	0	0	0	0
		**10.5**	0	0	0	0	3	0.28	0	0
		**11.5**	4	0.43	36	0.93	7	0.66	10	1.50
	**Boys**	**12.5**	94	5.73	923	11.27	297	14.11	152	13.52
		**13.5**	568	24.55	5,312	43.04	1,092	48.06	611	49.00
		**14.5**	1,594	71.29	10,934	82.83	1,846	85.54	1,018	85.98
		**15.5**	2,320	96.75	12,681	97.83	1,957	98.24	999	97.75
**2010**		**9.5**	1	0.13	8	0.22	0	0	2	0.71
		**10.5**	2	0.28	68	1.60	17	3.76	11	3.38
		**11.5**	11	2.04	1,242	27.08	193	46.17	152	49.51
	**Girls**	**12.5**	246	34.36	7,369	78.61	782	89.58	472	91.30
		**13.5**	681	71.76	12,798	95.25	1,228	98.32	569	97.10
		**14.5**	1,079	93.66	13,956	98.81	1,298	99.54	539	99.45
		**15.5**	1,748	99.32	14,943	99.58	1,250	99.60	487	99.39
		**9.5**	0	0	3	0.08	0	0	2	0.31
		**10.5**	0	0	7	0.19	2	0.21	0	0
		**11.5**	5	0.51	38	0.92	9	0.76	11	1.44
	**Boys**	**12.5**	149	9.28	1,328	16.49	381	17.73	193	17.55
		**13.5**	792	33.64	6,249	50.16	1,309	52.40	686	54.62
		**14.5**	1,542	74.89	10,756	83.92	1,915	86.57	1,036	87.43
		**15.5**	2,098	94.89	12,660	96.83	1,991	97.84	1,044	97.12
**2011**		**9.5**	1	0.11	5	0.14	1	0.24	2	0.75

		**10.5**	0	0.00	96	2.56	41	11.17	27	10.07
		**11.5**	13	2.26	1,344	29.06	218	48.88	170	51.20
	**Girls**	**12.5**	264	35.44	7,406	76.10	816	87.93	512	88.12
		**13.5**	741	74.62	12,595	95.05	1,161	97.15	556	96.70
		**14.5**	997	95.87	13,525	99.30	1,327	99.48	579	99.83
		**15.5**	1,590	99.62	14,900	99.67	1,233	99.68	438	99.55
**2012**	**Boys**	**9.5**	0	0	0	0	0	0	1	0.17
		**10.5**	1	0.16	4	0.13	0	0	1	0.16
		**11.5**	2	0.29	40	1.25	9	0.94	5	0.81
		**12.5**	105	7.15	1,399	17.52	421	17.76	219	17.35
		**13.5**	597	38.24	5,355	56.76	1,216	58.77	620	58.77
		**14.5**	1,130	75.18	9,196	87.56	1,700	86.82	925	88.69
		**15.5**	1,284	92.24	9,444	95.94	1,660	94.91	818	95.45
	**Girls**	**9.5**	0	0	10	0.32	2	0.53	0	0
		**10.5**	1	0.20	88	2.66	22	6.09	23	9.16
		**11.5**	16	4.16	907	24.28	172	41.35	127	42.33
		**12.5**	193	29.11	7,071	72.70	854	85.14	493	83.28
		**13.5**	483	73.85	9,985	92.10	969	95.94	479	94.66
		**14.5**	688	92.72	10,569	97.83	1,125	98.94	431	97.29
		**15.5**	1,076	98.81	11,525	98.64	1,111	99.11	369	98.40

Table S4 The trends in Median Age of Spermarche for boys and Median Age of Menarche for girls by different BMI levels from 2005 to 2012, Guangzhou, China.

	Year	Underweight		Normal-weight		Overweight		Obesity	
		**Median age**	**95% CI**	**Median age**	**95% CI**	**Median age**	**95% CI**	**Median age**	**95% CI**
**MAS (y)**	**2005**	13.76	13.70-13.82	13.45	13.37-13.42	13.35	13.28-13.42	13.24	13.15-13.35
	**2006**	13.63	13.57-13.68	13.31	13.23-13.30	13.30	13.23-13.38	13.16	13.03-13.28
	**2007**	13.63	13.56-13.70	13.30	13.23-13.30	13.19	13.11-13.26	13.25	13.15-13.35
	**2008**	13.85	13.80-13.91	13.46	13.40-13.45	13.27	13.21-13.34	13.32	13.23-13.42
	**2009**	13.78	13.69-13.86	13.39	13.31-13.38	13.22	13.13-13.30	13.36	13.28-13.45
	**2010**	13.88	13.82-13.95	13.54	13.42-13.50	13.42	13.35-13.49	13.47	13.38-13.56
	**2011**	13.82	13.75-13.88	13.47	13.40-13.47	13.39	13.32-13.46	13.25	13.13-13.38
	**2012**	13.87	13.78-13.96	13.45	13.38-13.45	13.48	13.42-13.55	13.38	13.26-13.50
**MAM (y)**	**2005**	12.88	12.79-12.97	12.11	12.02-12.09	11.69	11.52-11.85	11.57	11.35-11.79
	**2006**	12.86	12.76-12.96	12.06	12.00-12.07	11.58	11.43-11.74	11.51	11.30-11.72
	**2007**	12.94	12.85-13.04	12.09	12.04-12.11	11.68	11.53-11.83	11.54	11.34-11.75
	**2008**	13.08	12.99-13.18	12.04	11.99-12.06	11.52	11.38-11.66	11.52	11.33-11.72
	**2009**	12.82	12.71-12.93	12.10	12.05-12.12	11.76	11.62-11.90	11.59	11.42-11.77
	**2010**	12.89	12.78-12.99	12.11	12.05-12.12	11.70	11.56-11.83	11.73	11.56-11.90
	**2011**	12.74	12.63-12.85	12.05	12.02-12.08	11.70	11.58-11.83	11.60	11.44-11.76
	**2012**	13.02	12.90-13.13	12.20	12.16-12.23	11.83	11.71-11.97	11.73	11.54-11.93

Abbreviations: CI, confidence interval; MAS, Median Age of Spermarche; MAM, Median Age of Menarche.

Table S5 Logistic regression analysis of BMI levels and spermarcheal/menarcheal status from 2005 to 2012.

Variable	Spermarche		Menarche	
	Yes/No	OR (95% CI) *	Yes/No	OR (95% CI) *
**2005**				
**Normal-weight**	30,763/21,985	1.000	45,535/12,474	1.000
**Underweight**	6,586/6,799	0.491 (0.464-0.520)	5,322/3,721	0.152 (0.139-0.166)
**Overweight**	4,672/3,991	1.183 (1.103-1.269)	3,623/777	2.541 (2.181-2.960)
**Obesity**	2,267/2,114	1.307 (1.182-1.446)	1,603/532	2.549 (2.115-3.073)
**2006**				
**Normal-weight**	34,118/24,233	1.000	51,482/13,067	1.000
**Underweight**	7,356/7,124	0.593 (0.562-0.625)	4,976/3,478	0.160 (0.147-0.174)
**Overweight**	5,157/4,632	1.060 (0.994-1.129)	4,285/882	2.340 (2.031-2.697)
**Obesity**	2,434/2,566	1.052 (0.962-1.151)	2,076/556	2.577 (2.157-3.079)
**2007**				
**Normal-weight**	31,912/23,807	1.000	47,778/13,329	1.000
**Underweight**	6,551/6,808	0.551 (0.521-0.582)	4,412/3,174	0.144 (0.131-0.158)
**Overweight**	4,737/4,693	1.109 (1.039-1.184)	3,859/883	2.654 (2.299-3.063)
**Obesity**	2,274/2,570	1.142 (1.042-1.251)	1,909/613	2.675 (2.245-3.189)
**2008**				
**Normal-weight**	30,794/27,165	1.000	49,089/14,035	1.000
**Underweight**	5,114/6,820	0.470 (0.442-0.500)	3,762/3,037	0.126 (0.115-0.139)
**Overweight**	4,932/5,488	1.218 (1.141-1.301)	4,495/990	2.835 (2.473-3.249)
**Obesity**	2,446/3,157	1.176 (1.076-1.286)	2,117/680	2.794 (2.354-3.315)
**2009**				
**Normal-weight**	29,911/25,941	1.000	48,230/13,710	1.000
**Underweight**	4,939/6,352	0.484 (0.455-0.516)	4,018/3,000	0.151 (0.137-0.167)
**Overweight**	5,209/5,836	1.138 (1.068-1.213)	4,243/1,155	2.194 (1.913-2.516)
**Obesity**	2,820/3,467	1.256 (1.154-1.367)	2,155/769	2.779 (2.340-3.301)
**2010**				
**Normal-weight**	29,890/28,597	1.000	50,384/13,965	1.000
**Underweight**	4,580/6,627	0.483 (0.453-0.515)	3,768/2,856	0.146 (0.132-0.161)
**Overweight**	5,202/6,249	1.228 (1.152-1.310)	4,768/1,130	2.336 (2.045-2.669)
**Obesity**	2,790/3,838	1.258 (1.157-1.368)	2,232/815	2.495 (2.114-2.944)
**2011**				
**Normal-weight**	31,041/26,878	1.000	49,871/13,562	1.000
**Underweight**	4,586/6,233	0.521 (0.489-0.555)	3,606/2,926	0.162 (0.146-0.179)
**Overweight**	5,607/6,266	1.140 (1.073-1.211)	4,797/1,122	2.344 (2.066-2.659)
**Obesity**	2,972/3,750	1.196 (1.103-1.297)	2,284/760	2.481 (2.127-2.895)
**2012**				
**Normal-weight**	25,438/21,918	1.000	40,155/13,059	1.000
**Underweight**	3,119/4,677	0.433 (0.402-0.465)	2,457/2,204	0.221 (0.199-0.244)
**Overweight**	5,006/5,707	1.026 (0.961-1.094)	4,255/1,172	2.154 (1.915-2.422)
**Obesity**	2,589/,3465	1.054 (0.967-1.148)	1,922/792	1.989 (1.720-2.300)

Abbreviations: OR, odds ratio; CI, confidence interval. *Adjusted for students' age and districts of schools.
